# A Meta Analysis of Pancreatic Microarray Datasets Yields New Targets as Cancer Genes and Biomarkers

**DOI:** 10.1371/journal.pone.0093046

**Published:** 2014-04-16

**Authors:** Nalin C. W. Goonesekere, Xiaosheng Wang, Lindsey Ludwig, Chittibabu Guda

**Affiliations:** 1 Department of Chemistry and Biochemistry, University of Northern Iowa, Cedar Falls, Iowa, United States of America; 2 Department of Genetics, Cell Biology and Anatomy, University of Nebraska Medical Center, Omaha, Nebraska, United States of America; 3 Bioinformatics and Systems Biology Core, University of Nebraska Medical Center, Omaha, Nebraska, United States of America; Deutsches Krebsforschungszentrum, Germany

## Abstract

The lack of specific symptoms at early tumor stages, together with a high biological aggressiveness of the tumor contribute to the high mortality rate for pancreatic cancer (PC), which has a five year survival rate of less than 5%. Improved screening for earlier diagnosis, through the detection of diagnostic and prognostic biomarkers provides the best hope of increasing the rate of curatively resectable carcinomas. Though many serum markers have been reported to be elevated in patients with PC, so far, most of these markers have not been implemented into clinical routine due to low sensitivity or specificity. In this study, we have identified genes that are significantly upregulated in PC, through a meta-analysis of large number of microarray datasets. We demonstrate that the biological functions ascribed to these genes are clearly associated with PC and metastasis, and that that these genes exhibit a strong link to pathways involved with inflammation and the immune response. This investigation has yielded new targets for cancer genes, and potential biomarkers for pancreatic cancer. The candidate list of cancer genes includes protein kinase genes, new members of gene families currently associated with PC, as well as genes not previously linked to PC. In this study, we are also able to move towards developing a signature for hypomethylated genes, which could be useful for early detection of PC. We also show that the significantly upregulated 800+ genes in our analysis can serve as an enriched pool for tissue and serum protein biomarkers in pancreatic cancer.

## Introduction

Pancreatic cancer (PC) is a highly lethal malignancy, and patients with PC have a 5-year survival rate of less than 5% [1]. Thus, even though the incidence of breast cancer is estimated to be 5 times greater than PC, the annual death rates are comparable [2]. In nearly 95% of PC patients there is neither an associated family history of PC nor of diseases known to be associated with an increased risk of PC [3]. The lack of specific symptoms at early tumor stages, together with a high biological aggressiveness of the tumor and resistance to cytotoxic drugs all contribute to the high mortality rate of PC.

This study has been motivated by two reasons. The First is to contribute to the understanding of the fundamental disease etiology of PC by identifying novel candidate cancer genes in pancreatic cancer. The mutations found in a cancer cell genome have generally accumulated over the lifetime of the cancer patient and usually number between 1,000–10,000 [4]. For PC, exome sequencing has revealed that the average number of mutations in exons is about 60 [5]. Driver mutations [6] confer growth advantage on the cells carrying them and are positively selected for during the evolution of a cancer. It has been suggested that common adult epithelial cancers require the activation of between 5–20 such driver genes [7], [8]. The identification of driver mutations and the cancer genes that they alter has been a central aim of cancer research; so far, about 500 (2%) of the 22,000 protein-coding genes in the human genome are reported to show recurrent mutations in cancer with strong evidence that these contribute to cancer development [9] (http://www.sanger.ac.uk/genetics/CGP/Census/). However, studies in mice have suggested that more than 2,000 genes, when appropriately altered, may have the potential to contribute to cancer development [10] indicating that the search for cancer genes is far from over. A comprehensive treatment protocol for pancreatic cancer would require first, the identification of all the cancer genes, and next, the ability to modulate the function of these genes through therapeutic intervention. In recent years, the proteins altered by driver mutations have become targets for successful anticancer drug development [11]–[13].

The second impetus for this study comes from the paucity of biomarkers in PC [14], [15]. Improved screening for earlier diagnosis, through the detection of diagnostic and prognostic biomarkers, provides the best hope of increasing the rate of curatively resectable carcinomas. For example, analysis of sequence data has suggested that the time frame from the initiation of the pancreatic tumor to the development of metastatic subclones could be more than ten years [16]. Though many serum markers has been reported to be elevated in patients with pancreatic cancer, so far, most of these markers have not been implemented into clinical routine due to low sensitivity or specificity [14] with the exception of CA 19-9 [17].

Meta-analysis of microarray datasets consists of using statistical techniques to combine results from several studies in order to increase statistical power and generalizability compared with any single study [18]. This addresses, to some extent, the issues of biological and technical variations, which can have a significant effect on microarray measurements [19]. The previous meta-analysis of microarray datasets on PC was conducted nearly a decade ago by Grutzman and colleagues [20], and the analysis was limited to a few thousand genes.

In this study, we examined the differential gene expression patterns that are replicated across datasets, to create a ranked list of genes overexpressed in PC. We focused our attention only on genes that are overexpressed, since about 80% of cancer genes are dominant acting [4] through either overexpression or constitutive activation of gene product. In this study, we have detected hundreds of genes that were significantly upregulated in pancreatic cancer. The list of overexpressed genes include genes that have not been previously associated with PC as well as new members of gene families that have been associated with PC. We have also identified tens of kinase-encoding genes overexpressed in pancreatic cancer, which are potential therapeutic targets for PC. In this study, we are also able to move towards developing a signature for hypomethylated genes, which could be useful for early detection of PC. We also find that about a third of the putative protein serum biomarkers thus far identified for PC are, in fact, significantly overexpressed in our analysis, indicating that our results could serve as a resource for further experimental studies, in the quest for effective biomarkers for PC.

## Materials and Methods

### Pancreatic cancer microarray datasets

Nine pancreatic cancer datasets in the Oncomine database [21] that contained a differential analysis of pancreatic cancer vs. normal samples, were included this study (Table 1). Oncomine [21] is the most comprehensive cancer-specific database, currently containing 628 datasets investigating 35 tumor types (Oncomine 4.4 Research Edition). The advantage of using datasets from Oncomine is that prior to inclusion in Oncomine, the microarray datasets (obtained from public resources such as Stanford Microarray Database and the NCBI Gene Expression Omnibus or literature sources) are reviewed by a panel of experts to ensure that they meet certain quality standards [22].

**Table 1:** Pancreatic Cancer Microarray Datasets Included in the Study.

Dataset Name*	Cancer Type	Genes**	Platform	Dataset Summary*
Badea Pancreas	Pancreatic Ductal Adenocarcinoma	19,574	Human Genome U133 Plus 2.0 Array	Paired pancreatic ductal adenocarcinoma (n = 39) and normal pancreas (n = 39) samples from 36 patients were analyzed; three patients were analyzed in duplicate.
Buchholz Pancreas	Pancreatic Ductal Adenocarcinoma	15,725	Human Genome Oligo-Set-Version 2.0 (Operon, Germany)	Eight (8) pancreatic ductal adenocarcinoma and 6 normal pancreatic duct samples were analyzed.
Buchholz Pancreas	Pancreatic Intraepithelial Neoplasia	15,736	Human Genome Oligo-Set-Version 2.0 (Operon, Germany)	Twenty-four (24) pancreatic intraepithelial neoplasia and 6 normal pancreatic duct samples were analyzed.
Grutzmann Pancreas	Pancreatic Ductal Adenocarcinoma	17,782	Human Genome U133A Array, Human Genome U133B Array	Fourteen (14) microdissected pancreatic ductal adenocarcinoma and 11 normal pancreatic duct samples were analyzed. Sample data includes type, age, grade, TNM stage, and sex.
Ishikawa Pancreas	Pancreatic Ductal Adenocarcinoma	17,782	Human Genome U133A Array, Human Genome U133B Array	Twenty-four (24) pancreatic ductal adenocarcinoma and 25 normal pancreatic duct samples were analyzed. Sample data includes type, age, atypical cell proportion, clinical stage, cytological grade, and sex.
Iacobuzio-Donahue Pancreas 2	Pancreatic Ductal Adenocarcinoma	14,361	Non standard	Fourteen (14) pancreatic carcinoma cell lines, 17 primary pancreatic ductal adenocarcinoma samples of various histologies, and 5 normal pancreas samples were analyzed.
Logsdon Pancreas	Pancreatic Adenocarcinoma	5,338	HumanGeneFL Array	Ten microdissected adenocarcinoma, 7 pancreatic cancer cell lines, 5 pancreatitis, and 5 normal pancreas samples were analyzed. Sample data includes type and cell line name.
Pei Pancreas	Pancreatic Ductal Adenocarcinoma	19,574	Human Genome U133 Plus 2.0 Array	Thirty-six (36) pancreatic carcinoma and 16 paired normal samples, for a total of 52 samples, were analyzed. Sample data includes age and sex.
Segara Pancreas	Pancreatic carcinoma	12,684	Human Genome U133A Array	Eleven (11) pancreatic adenocarcinoma samples and six (6) adjacent normal pancreas samples from 12 patients were analyzed.

*As identified by the Oncomine database.

**Number of genes probed.

### Initial screening of microarray datasets

Prior to combining microarray datasets from different sources, a further quality check was performed on the datasets using the program Venn Mapper [23]. Venn mapper can identify significant similarities between heterologous microarray datasets, by comparing the overlap of differentially expressed genes and calculating a statistical significance using z-values. Briefly, a 2-fold cutoff is used to determine the upregulated genes in a microarray dataset. A list of upregulated genes is established for each microarray, and all pair-wise (except self comparisons) combinations of lists are compared for matching gene-identity (i.e. HUGO gene names). The number of genes commonly upregulated, *R_observed_*, in any two experiments is determined, and a z-value is calculated to determine whether this number is statistically significant. For two microarrays A and B, the z-value is calculated as follows:
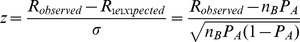

*R = number of genes upregulated in both A and B*

*n_B_ = Total number of genes upregulated in B*

*P_A_ = Probability of a gene being upregulated in A*

Microarrays were clustered based on z-value profiles, and any outliers were identified, and omitted from further analysis. An absolute z-value of >1.96 is equivalent to a p-value of <0.05.

### Obtaining ranked lists of upregulated genes

To identify differentially expressed genes across multiple datasets, we employed a non-parametric ‘rank product’ method implemented in the RankProd package [24], [25]. RankProd is a statistically rigorous but biologically intuitive algorithm, which has been shown to be robust against noise in microarray data [26], [27]. RankProd has been shown to have higher sensitivity and specificity compared to other types of meta-analytic tools for microarrays [28]. A list of upregulated genes are selected based on a conservative estimation of the percentage of false positive predictions (pfp), which is also known as the false discovery rate. As recommended, a pfp value of <0.15 [25] was used to set the threshold for genes that are significantly upregulated.

## Results and Discussion

### Congruency between microarray datasets

The program Venn Mapper [23] was used to perform an initial screening, to determine any broad inconsistencies that exist between the microarray datasets. Analysis was carried out on nine different datasets, and all-to-all pairwise z-values are given in Table 2. Two outliers were identified by this method, namely, Buchholz Pancreas (Pancreatic Ductal Adenocarcinoma) and Buchholz Pancreas (Pancreatic Intraepithelial Neoplasia). The low z-values associated with these datasets indicate a lack of significant correlation between upregulated genes in these datasets, when compared with other datasets. Hence, these two datasets were omitted from further analyses. While we are uncertain about the source of this incongruency, we note that the Buchholz datasets were the only datasets obtained without the use of standard (commercially available) platforms. Another dataset, Logsdon Pancreas, was also omitted due to the low number of genes in the dataset (5,338, compared to an average of 16,652 genes for the rest of the data (Table 1)).

**Table 2:** Pairwise z-values* Indicating Congruency between Upregulated Genes.

Name	Badea^1^	Buch_DAC^2^	Buch_Intra^3^	Grutzmann^4^	Iacobuzio^5^	Ishikawa^6^	Logsdon^7^	Pei^8^
Buch_DAC	**0.6**							
Buch_Intra	**0.2**	17.6						
Grutzmann	9.7	**−0.2**	**−0.6**					
Iacobuzio	9.7	**−0.1**	**−0.6**	5.2				
Ishikawa	4.4	**−0.6**	**−0.6**	7.8	2.1			
Logsdon	14.9	**0.1**	**−0.9**	8.7	9.5	**1.3**		
Pei	20.8	**−0.6**	**−1.5**	12.4	12.3	4.2	13.9	
Segera^9^	17.5	**0.7**	**0.4**	7.2	8.2	6.1	11.5	12.7

*A ***z*** **-value of >1.96 indicates a** ***p*** **-value of <0.05.**

1 Badea Pancreas (Pancreatic Ductal Adenocarcinoma).

2 Buchholz Pancreas (Pancreatic Ductal Adenocarcinoma).

3 Buchholz Pancreas (Pancreatic Intraepithelial Neoplasia).

4 Grutzmann Pancreas (Pancreatic Ductal Adenocarcinoma).

5 Iacobuzio-Donahue (Pancreatic Adenocarcinoma).

6 Ishikawa Pancreas (Pancreatic Ductal Adenocarcinoma).

7 Logsdon Pancreas (Pancreatic Adenocarcinoma).

8 Pei Pancreas (Pancreatic Ductal Adenocarcinoma).

9 Segera Pancreas (Pancreatic Carcinoma).

Below, we organize our results and discussion into four discrete sections that include identification of upregulated genes, functional analysis of upregulated genes, identification of a genetic signature for hypomethylation in PC, and identification of potential tissue, serum and matrix metalloproteinase biomarkers in PC.

### Identification of upregulated genes

RankProd [24] yields a list of genes ranked by percentage of false positive prediction (pfp) value (see methods). Of the 5590 genes that were upregulated by at least two fold, 827 genes are found to be significantly upregulated when using a pfp threshold of <0.15 [25] (Table S1).

Table 3 provides a list of the top twenty-five ranked genes using the RankProd program. As expected, most genes have well-established associations with pancreatic and other cancers. Some well-known examples include MUC4 [29], CEACAM5/6 [30], S100P [31], CLDN18 [32], KRT19 (CK19) [33] and COLA1/2 [34]. There are, however, some notable exceptions such as AHNAK2, CTHRC1, IGHG3 and EPPK1, which do not have a known role in cancer. Hence, these genes can be potential new leads for cancer genes, and are discussed next.

**Table 3:** A List of the 25 Most Highly Ranked Upregulated Genes in Pancreatic Cancer.

Gene*	Gene Function
AHNAK2	Unknown; a component of the costameric network
CDH3	A calcium-dependent cell adhesion molecule.
CEACAM5	Cell surface glycoprotein that plays a role in cell adhesion and in intracellular signaling; binds with another CEACAM to function.
CEACAM6	A cell adhesion molecule; mediates cell adhesion by binding with another CEACAM (−1, −5, and −6 are most common).
CLDN18	Plays a major role in tight junction-specific obliteration of the intercellular space, through calcium-independent cell-adhesion activity
COL11A1	This gene encodes one of the two alpha chains of type XI collagen, a minor fibrillar collagen.
COL1A1	Type 1 collagen is a fibril forming collagen found in most connective tissue; alpha chain one.
COL1A2	Type 1 collagen is a fibril forming collagen found in most connective tissue; alpha chain two.
CTHRC1	May play a role in the cellular response to arterial injury through involvement in vascular remodeling. (secreted)
CTSE	A gastric aspartyl protease that functions as a disulfide-linked homodimer.
EPPK1	Unknown. May play a role in supporting the intermediate filaments
FN1	Fibronectin is involved in cell adhesion and migration processes including embryogenesis, wound healing, blood coagulation, host defense, and metastasis. (secreted)
GPRC5A	Unknown. May be involved with the interaction between retanoic acid and the G protein sigaling pathway.
IGHG3	Unknown; Immunoglobulin heavy chain gamma 3
KRT19	Involved in the organization of myofibers. Together with KRT8, helps to link the contractile apparatus to dystrophin at the costameres of striated muscle.
MMP11	Weakly degrades structural proteins of the ECM.
MUC4	Mucins are glycoprotein that play a role in the protection of epithelial cells. Implicated in renewal and differentiation.
OLFM4	An antiapoptotic factor that promotes tumor growth and is an extracellular matrix glycoprotein that facilitates cell adhesion.
POSTN	Induces cell attachment and spreading and plays a role in cell adhesion.
S100P	S100 proteins are involved in the regulation of a number of cellular processes such as cell cycle progression and differentiation.
SERPINB5	Unknown. Exhibits no serine protease inhibitory activity functions as a tumor suppressor of mammary tumors
SLC6A14	A member of the solute carrier family; transports both neutral and cationic amino acids
VCAN	This protein is involved in cell adhesion, proliferation, migration and angiogenesis and plays a central role in tissue morphogenesis and maintenance
THBS2	A disulfide-linked homotrimeric glycoprotein that mediates cell-to-cell and cell-to-matrix interactions
COL3A1	Pro-alpha1 chain of type III collagen, a fibrillar collagen that is found in extensible connective tissue.

*Gene names are given according to the HUGO Gene Nomenclature Committee (HGNC).

AHNAK2 is a significantly upregulated gene in PC (175-fold), but has not been directly associated with any cancer, to our knowledge. The mRNA is reported [35] to be alternatively spliced to produce three isoforms, and the canonical sequence is inferred to be targeted to the nucleus. The AHNAK family of scaffold PDZ proteins consists of two large proteins (600–700 kD), AHNAK (desmoyokin) and AHNAK2 [36]. AHNAK has been associated with several muscular diseases, including cardiomyopathy and limb-girdle muscular dystrophy, and this effect is believed to be mediated through its association with the β-subunit of cardiac Ca(v) calcium channel [37]. AHNAK & AHNAK2 have also been shown to be components of the costameric network, associated with linking of the extracellular matrix to the cytoplasmic microfilament system [38]. Experiments on metastatic human tumor cell lines [39] have shown that knockdown of AHNAK resulted in pseudopod retraction, inhibition of cell migration and reversion of mesenchymal-epithelial transition (MET). It is likely that AHNAK and AHNAK2 were both affected by these knockdown experiments. Our results suggest that the family of AHNAK proteins, particularly AHNAK2, merit experimental scrutiny regarding their possible role in carcinogenesis, especially in PC.

CTHRC1 (collagen triple helix containing 1) is a 30 kD secreted protein that has the ability to inhibit collagen matrix synthesis, and is highly expressed during skin wound healing. Tissue repair and carcinogenesis are linked [40] and CTHRC1 has been associated with a variety of tumors including melanoma [41], breast cancer [42], colorectal cancer [43] and most recently, gastric cancer [44]. However, there has only been one report that links CTHRC1 with PC, where higher expression of CTHRC1 was observed in a screen of solid tumor cell lines including PC [41]. There is evidence that CTHRC1 expression is associated with cancer tissue invasion and metastasis in breast cancer [42] and gastric cancer [44]. Given the high level of upregulation of CTHRC1 (>1,000-fold) that was observed in this study, we hypothesize CTHRC1 to be an excellent candidate for experimental evaluation as a potential biomarker for PC.

**Figure 1:**
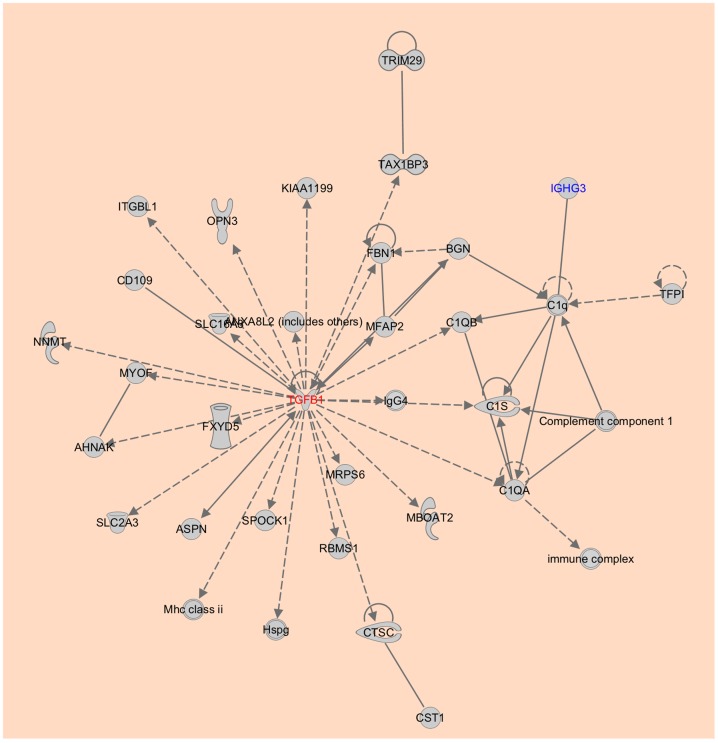
Top scoring network associated with the upregulated genes in PC. TGFB1 forms a hub node in the network. IGHG3 (highlighted in blue color) is one of the top twenty-five genes that is potentially important for pancreatic cancer.

IGHG3 (Immunoglobulin heavy constant γ-3) is a secreted antigen binding protein not previously implicated in pancreatic cancer. Our analysis (see next section) indicates that PC is associated with dysfunction of the immune system. IGHG3 is also a component of the top network associated with the cohort of 827 overexpressed genes, which is shown in Figure 1.

Epiplakin belongs to the plakin family of cytolinker proteins that are associated with the junctional complexes and the cytoskeleton. Epiplakin is rather an unusual plakin in that it consists solely of plakin repeats organized into 13 plakin repeat domains (PRD's) and does not contain a plakin domain characteristic of other plakins. There is evidence to suggest that Epiplakin associates with keratin networks during wound healing [45].

### Functional analysis of the upregulated genes

We identified important functions, networks, and pathways relevant to the 827 significantly upregulated genes using IPA (www.ingenuity.com). A comprehensive analysis of the 827 upregulated genes is shown in Table S1.

**Figure 2:**
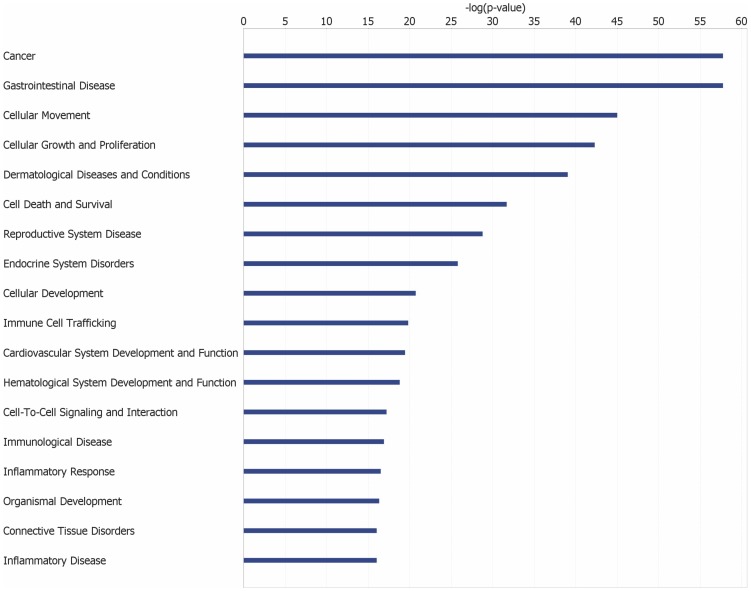
Important biological functions and diseases associated with genes upregulated in PC.

**Figure 3:**
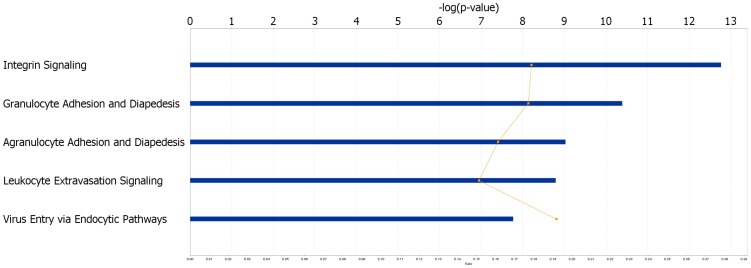
The five most significant pathways associated with genes upregulated in PC are related to inflammation and immune response.

The most significant biological functions associated with the 827 upregulated genes are cellular movement, cellular growth and proliferation, cell death and survival, cellular development and cell-to-cell signaling and interaction (Figure 2, Table S2). Dysregulation of these functions are associated with cancer and metastasis, reiterating the importance of this geneset to PC. A pathway analysis provided insights into some of the molecular mechanisms important in PC. The five most significant pathways associated with the 827 upregulated genes included integrin signaling (p-value = 1.72×10^−13^), also observed by Grutzmann et al. [20], granulocyte adhesion and diapedesis (p-value = 4.08×10^−11^), agranulocyte adhesion and diapedesis (p-value = 9.43×10^−10^), leukocyte extravasation signaling (p-value = 1.62×10^−9^), and virus entry via endocytic pathways (p-value = 1.71×10^−8^) (Figure 3, Table S3). These results indicated that PC is significantly associated with inflammation and immune mechanisms. In fact, it has been shown that cancer immunosuppression often favors tumor progression and metastasis by constituting an immunosuppressive network in which several tumor-derived soluble factors such as interleukin-10, transforming growth factor beta (TGFB) and vascular endothelial growth factor play central roles [46]. In the top network identified, TGFB1 is the hub gene (Figure 1). TGFB1 encodes a member of the TGFB family of cytokines, which are multifunctional peptides that regulate proliferation, differentiation, adhesion, migration, and other functions in many cell types. This gene has been shown to be frequently upregulated in tumor cells, and is an important target for cancer therapy [47]–[51].

**Figure 4:**
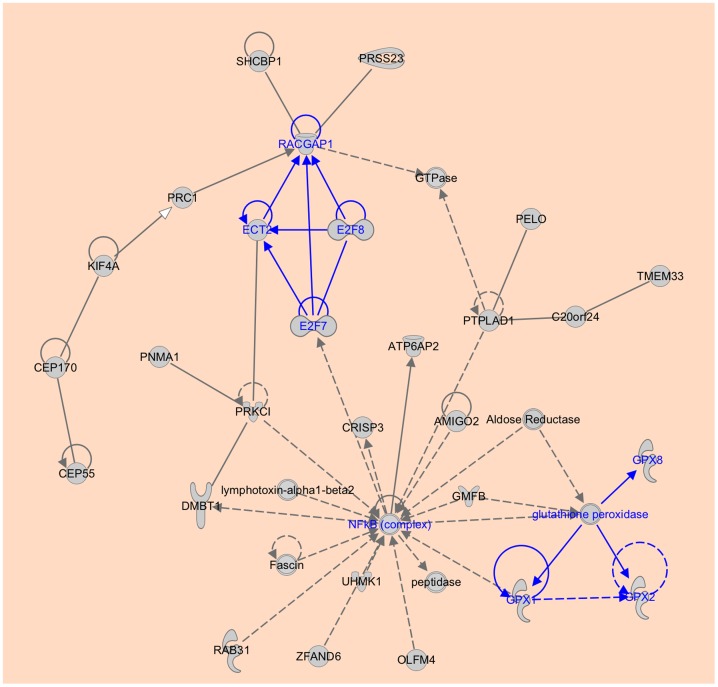
The second most significant network associated with the upregulated genes in PC. A major hub node NF-κB complex, and two new regulatory modules are highlighted in blue color. One module is made up of two E2F family genes (E2F7, E2F8), ECT2 and RACGAP1; and another module is made up of several GPX family genes.

The second most significant network associated with the upregulated genes is involved in cell cycle, cellular movement, and cancer (Figure 4). In this network, NF-κB complex acts as a major hub, which functions as a regulator of genes that control cell proliferation and cell survival. Incorrect regulation of NF-κB has been linked to cancer, inflammatory and autoimmune diseases [52], [53]. This network again suggests that PC could be closely correlated with immunological disorder [54], [55]. Upregulated NF-κB turns on the expression of genes that keep the cell proliferating, and protect the cell from conditions that would otherwise cause it to die via apoptosis. In fact, it has been shown that NF-κB is constitutively active in various types of human tumors [56]–[60]. In addition, there are two interesting regulatory modules identified in this network. The first module is made up of two E2F family genes (E2F7, E2F8), ECT2 and RACGAP1. These genes form autoregulatory loops, and regulate each other. Notably, the three genes E2F7, E2F8 and ECT2 constitutively regulate RACGAP1, which binds to Rho GTPases (Figure 4), suggesting that this module functions in the regulation of cytokinesis in a cell cycle-dependent manner. Another module involves the glutathione peroxidase (GPX) family genes that encode an enzyme family with peroxidase activity, whose main biological role is to protect the organism from oxidative damage. Upregulation of GPX family genes may be associated PC and other cancers [61]–[64], suggesting an important link between oxidatively-induced DNA damage and cancer development.

### Identification of the upregulated kinase-encoding genes

Furthermore, we extracted the genes encoding protein kinases from the 827 upregulated genes. Table 4 presents the 26 kinase-encoding genes. It has been known that many kinase-encoding genes are upregulated in cancer, and development of anticancer drugs that inhibit overexpression of protein kinases has been an active area of research. In fact, effective drugs have already been developed to target some of the protein kinases. For example, CDK1 encodes a member of the Ser/Thr protein kinase family, which is a catalytic subunit of the highly conserved protein kinase complex known as M-phase promoting factor. The protein plays a key role in G1/S and G2/M phase transitions of eukaryotic cell cycle, and the phosphorylation and dephosphorylation of this protein play important regulatory roles in cell cycle control [65]. Some CDK1 kinase inhibitors have been developed for clinical or experimental purposes - AZD 5438, (R)-CR8, (R)-DRF053 dihydrochloride, Kenpaullone, NU 2058, and Ro 3306 (Tocris Bioscience, www.tocris.com), and ZK 304709 and Terameprocol [66].

**Table 4:** Twenty-six Kinase-encoding Genes Upregulated in Pancreatic Cancer.

Gene*	Description
ACVR1	Activin A receptor, type I
BUB1	Budding uninhibited by benzimidazoles 1 homolog (yeast)
BUB1B	Budding uninhibited by benzimidazoles 1 homolog beta (yeast)
CDK1	Cell division cycle 2, G1 to S and G2 to M
DYRK2	Dual-specificity tyrosine-(Y)-phosphorylation regulated kinase 2
EPHA4	EPH receptor A4
IRAK3	Interleukin-1 receptor-associated kinase 3
LCK	lymphocyte-specific protein tyrosine kinase
LYN	V-yes-1 Yamaguchi sarcoma viral related oncogene homolog
MAP4K4	Mitogen-activated protein kinase 4
MELK	Maternal embryonic leucine zipper kinase
MET	Met proto-oncogene (hepatocyte growth factor receptor)
MST1R	Macrophage stimulating 1 receptor (c-met-related tyrosine kinase)
MST4	Serine/threonine protein kinase MST4
NEK2	NIMA (never in mitosis gene a)-related kinase 2
NUAK1	NUAK family, SNF1-like kinase, 1
PBK	PDZ binding kinase
PRKCI	Protein kinase C, iota
PTK6	PTK6 protein tyrosine kinase 6
STK17B	Serine/threonine kinase 17b
STK24	Serine/threonine kinase 24 (STE20 homolog, yeast)
STYK1	Serine/threonine/tyrosine kinase 1
TNIK	TRAF2 and NCK interacting kinase
TRIB2	Tribbles homolog 2 (Drosophila)
TTK	TTK protein kinase
UHMK1	U2AF homology motif (UHM) kinase 1

*Gene names are given according to the HUGO Gene Nomenclature Committee (HGNC).

LCK is a tyrosine-protein kinase that is found inside lymphocytes of the immune system, and involved in immune signaling pathways. Dasatinib, a small-molecule protein tyrosine kinase inhibitor and anticancer drug, can inhibit LCK activity in T-cell activation and proliferation [67], [68]. MET is a proto-oncogene that encodes the hepatocyte growth factor receptor protein [69], which possesses tyrosine-protein kinase activity. Abnormal upregulation of MET in cancer often correlates with poor prognosis by triggering tumor growth, angiogenesis that supply the tumor with nutrients, and metastasis. It has been revealed that the MET pathway is one of the most frequently dysregulated pathways in human cancer [70]. A substantial number of MET inhibitors have been studied in clinical trails like AMG-458 (Amgen), PF-04217903 (Pfizer), MK-2461(Merck), ARQ197 (ArQule) etc. [71].

TTK encodes a dual specificity protein kinase with the ability to phosphorylate tyrosine, serine and threonine. TTK kinase is associated with cell proliferation and is essential for the proper attachment of chromosomes to the mitotic spindle. Inhibition of TTK kinase has been shown to correlate with cell death caused by chromosomal missegregations [72]. Several TTK kinase inhibitors have been reported in the literature – Reversine [73], NMS-P715 [74], and MPS1-IN-1 [75].

For some other tyrosine-protein kinases such as LYN, Dasatinib is an effective inhibitor [76]. Of the 26 kinase-encoding genes we identified, some genes have been identified as very promising anticancer targets. For example, BUB1 encoding the mitotic checkpoint serine/threonine-protein kinase is critical in the establishment of the mitotic spindle checkpoint and chromosome congression. It has been shown that disturbed mitotic checkpoints are a common feature of many human cancers [77]. However, BUB1 expression levels depend on the localization of tumors and their severity [78]. Downregulation of BUB1 resulted in more sarcomas, lymphomas and lung tumors, whereas upregulation of BUB1 caused sarcomas and tumors in the liver [78]. Our result shows that PC is related to the upregulation of BUB1 and we speculate that development of BUB1 inhibitors could provide a new approach to tackling PC.

To sum up, some of the 26 significantly upregulated protein kinase genes in PC could be viable new therapeutic targets for PC. In fact, for the tyrosine-protein kinase genes such as LCK, MET and LYN, which have been found to be frequently overexpressed in human cancer including PC [79], effective tyrosine-protein kinase inhibitors such as Dasatinib, Imatinib, Gefitinib, Erlotinib, and Sunitinib have been developed for anticancer chemotherapy [80].

### Towards a genetic signature for hypomethylation in pancreatic cancer

Aberrant hypermethylation of promoter CpG islands is tightly associated with gene silencing, whereas hypomethylation can lead to the upregulation of genes. A recent review [81], discusses genes that have been found to be hypomethylated in PC. With reference to this gene set, we do find a strong correlation between hypomethylation and upregulation; specifically, seven of nine genes mentioned in this review (SERPINB5, CLDN4, SFN, S100P, S100A4, MSLN, and PSCA) are significantly upregulated, with SERPINB5, SFN, S100P, and PSCA being among the 100 most upregulated genes in our analysis (Table S1).

A comprehensive study on aberrant methylation in PC has been performed by Tan et al. [82], who profiled 1505 CpG sites across 807 genes. Initial investigations yielded a list of 63 genes with CpG site hypomethylation and increased mRNA expression. Somewhat unexpectedly, the authors also found a similar number of genes with CpG site hypomethylation and decreased mRNA expression. Upon further experimentation, 35 of the 63 genes were identified by the authors as candidate genes that are regulated by hypomethylation in PC. We find that eight of the 35 candidate genes (ID1, MMP7, MST1R, NBL1, PHLDA2, PLAT, PLAUR and SFN), and a further 8 (IL8, SPP1, CLDN4, MMP1, ARHGDIB, NQO1, ITGB4, SERPINB5, and TFF1) from the original list of 63 genes are also significantly upregulated in our study.

To summarize, twenty-two genes (MUC4, SERPINB5, CLDN4, SFN, TFF1, S100P, S100A4, MMP1, MMP7, MSLN, PSCA, ID1, MST1R, NBL1, PHLDA2, PLAT, PLAUR, IL8, SPP1, ARHGDIB, NQO1, and ITGB4) are significantly upregulated in our analysis, and there is experimental evidence [81], [82] to suggest that this upregulation is due to hypomethylation. Thus, these genes will contribute towards a growing list of candidates including MUC4 [83] that describe a putative genetic signature for hypomethylation in pancreatic cancer (Table 5). Such a genetic signature could prove to be useful in the early detection of PC, in a manner analogous to the clinical use of aberrant methylation of CCND2 [84] in PC. Since there is an emerging consensus that ‘epigenetic chaos’ promoted changes in gene expression and, ultimately, leads to cancer [85], it is quite likely that many of the genes found to be significantly upregulated (Table S1) are hypomethylated in PC. Of the 22 genes, IPA analysis reveals that 11 genes have a known association with PC (Table 5).

**Table 5:** A Putative Genetic Signature of Hypomethylated Genes in Pancreatic Cancer.

Gene	pfp value	Log (2) value	Reference*
MUC4$	0.000	6.28	Zhu et al., 2011 [83]
SERPINB5	0.000	8.34	Sato et al., 2003 [110]; Fitzgerald et al., 2003 [111]; Ohike et al., 2003 [112]
CLDN4	0.103	2.67	Sato et al., 2003 [110], Tan et al., 2009 [82] (**Gastric sarcoma** Kwon et al., 2011 [113])
SFN	0.000	7.91	Sato et al., 2003 [110], Tan et al., 2009 [82] (**Lung adenocarcinoma** Shiba-Ishii et al., 2012 [114])
TFF1$	0.001	7.27	Tan et al., 2009 [82] (**Prostrate cancer** Vestergaard et al., 2010 [115])
S100P$	0.000	24.25	Sato et al., 2003 [110]
S100A4	0.004	3.12	Rosty et al., 2002 [116]
MMP1	0.003	4.63	Tan et al., 2009 [82]
MMP7$	0.011	4.07	Tan et al., 2009 [82]
MSLN$	0.026	4.80	Sato et al., 2003 [110] (**Mesothelioma** Nelson et al., 2011 [117])
PSCA$	0.001	7.78	Sato et al., 2003 [110]
ID1$	0.151	2.06	Tan et al., 2009 [82]
MST1R	0.025	3.40	Tan et al., 2009 [82]
NBL1	0.035	2.82	Tan et al., 2009 [82]
PHLDA2	0.000	5.22	Tan et al., 2009 [82] (**Osteosarcoma** Li et al., 2008 [118])
PLAT$	0.014	3.34	Tan et al., 2009 [82]
PLAUR$	0.014	3.18	Tan et al., 2009 [82]
IL8	0.007	4.21	Tan et al., 2009 [82] (**Colorectal adenocarcinoma** Dimberg et al., 2012 [119])
SPP1$	0.044	2.14	Tan et al., 2009 [82] (**Liver fibrosis** Komatsu et al., 2012 [120])
ARHGDIB	0.021	2.40	Tan et al., 2009 [82]
NQO1	0.000	6.19	Tan et al., 2009 [82]
ITGB4$	0.014	3.06	Tan et al., 2009 [82]

*References for hypomethylation in other cancers are given in parenthesis.

$ IPA analysis indicates a known association with PC.

### Potential biomarkers among upregulated genes

#### Tumor tissue protein biomarkers

An observation often reported in literature is the discrepancy between the level of expression of a protein and that of its transcript for a given type of cell [86]. Nonetheless, we find about 70% of thirty two tumor tissue protein biomarkers identified in two recent reviews [87], [88] were found to be upregulated >2-fold in our analysis. Among those significantly upregulated (pfp<0.15) were a cluster of genes associated with the actin microfilament, lGAlS1 (galectin-1), ACTN4 (actinin-4), PLS1 (plastin-1), TPM2 (tropomyosin β), CFL1 (cofilin-1), ENO1 (α-enolase), and MSN (moesin). Most of these proteins are known actin-binding proteins that can modulate the actin microfilament, or modulate its environment with the plasma membrane.

Other suggested tumor tissue protein biomarkers [87], [88] significantly upregulated in our analysis include SFN, AGR2, LGALS1, LGALS3, THBS2, & TGFB1, and four members of the S100 family, S100A6, S100A10, S100A11, and S100A2 [89]. We find three additional members of the S100 family, S100A4, S100A16 and S100P were also significantly upregulated (Table S1). The S100 family of low molecular weight calcium binding proteins have strong associations with cancer [90], and several of them have been used as markers in melanoma and other cancers. It should be noted that S100P is one of the most upregulated genes in our analysis (>×10^6^). It has recently been proposed that S100P be used as a protein biomarker for intraductal papillary mucinous neoplasms (IPMN) of the pancreas [91], and for pancreatic adenocarcinoma [92].

#### Serum protein biomarkers

Early diagnosis of pancreatic cancer is essential in order to improve the poor prognosis associated with PC. Serum biomarkers offer a very attractive and non-invasive solution, and are thus highly sought after [14]. However, there is a paucity of serum biomarkers for PC [15], with the carbohydrate biomarker CA 19-9 being the most widely used.

Since serum protein biomarkers such as CA-125 may be cleaved and released in PC [93] a correlation between serum biomarkers and mRNA expression is not necessarily expected (though in the case of CA-125, there is evidence that it is overexpressed as well [93]). Nevertheless, we sought to investigate whether any of the proposed serum protein biomarkers in the recent literature [3] were upregulated in pancreatic cancer at the level of mRNA. Somewhat to our surprise, we found that about one-third of the corresponding genes, C3, B2M, C1QB, CD9, TIMP1, PGK1, SERPINA1, APOE, AGR2, APOC1 & SPP1, were significantly upregulated in our analysis. These results indicate our corhort of 827 significantly upregulated genes also represent an enriched pool of candidate serum protein biomarkers. The commercial availability of many human antibodies raises the intriguing possibility of performing a systematic screen of serum, to detect for protein products of significantly upregulated genes in our analysis. While individual biomarkers may suffer from issues of sensitivity and specificity [14], the promise is that with a large number of biomarkers, distinctive signatures are likely to emerge, that correlate with diagnosis and prognosis.

#### Matrix metalloproteinase biomarkers

Matrix metalloproteases represent the most prominent family of proteinases associated with tumorigenesis [94]. In our analysis, we found that seven matrix metalloproteases (MMPs) and six proteases from a related family “a disintegrin and metalloprotease” (ADAMs) to be significantly upregulated (Table 6). Three of these (MMP9, ADAM9 and ADAM10) were also found to be upregulated by Grutzman *et al.* [20].

**Table 6:** Matrix Metalloproteinases Upregulated in Pancreatic Cancer.

Gene	pfp value	Log (2) value
MMP11	0.00	15.71
MMP12	0.00	4.49
MMP1	0.00	4.63
MMP7	0.01	4.07
MMP2	0.03	2.79
MMP28	0.07	2.37
MMP9	0.15	1.87
ADAM8	0.01	2.91
ADAM9	0.05	2.56
ADAM12	0.06	2.34
ADAMTS6	0.07	2.97
ADAMTS12	0.08	2.54
ADAM28	0.10	2.50
ADAM10	0.16	1.67

Matrix metalloproteases are a family of zinc-dependent proteases that have the capacity to degrade virtually every component of the extracellular matrix (ECM). Tumor cells overexpress these proteases in order to degrade the basement membrane and invade the surrounding tissue. This activity is also required for the intravasation and extravasation events in metastasis. MMP substrates also include non-ECM molecules, ranging from growth factor precursors and cell surface adhesion molecules to angiogenic inhibitor precursors [95]. MMPs have also been implicated in the epithelial to mesenchymal transition (EMT) [96]. While MMPs have well-recognized roles in the late stage of tumor progression, invasion, and metastasis, emerging evidence suggests that the role of MMPs in tumorigenesis is more complex [97].

One of the more promising and exciting applications of MMPs in human cancers is as potential cancer biomarkers, both diagnostic and prognostic. MMP-2, MMP-7 and MMP-9 are among the most well studied matrix metalloproteases in PC [98]. MMP-9 expression has been linked to worse prognosis, and it also significantly correlated with tumor expression and distant metastasis [99]. Active MMP-2 levels are upregulated in the pancreatic juice of patients with cancer (100%) as compared with patients with chronic pancreatitis (2%) or normal controls (0%) [100] Similarly, plasma as well as tumor tissues from patients with pancreatic ductal adenocarcinoma have significantly elevated MMP-7 levels, which may predict shortened survival of patients [101].

As expected, MMP-2, MMP-7 and MMP-9 are all significantly upregulated in our study. However, another matrix matalloprotease, MMP-11, is the most highly upregulated MMP, with an average >10,000 fold overexpression in PC. MMP-11 induction in adipose tissue has been linked to cancer progression [102] and MMP-11 has been associated with tumor progression in pulmonary cancer [103], head and neck carcinoma [104] and breast carcinoma [105]. MMP-11 is known to cleave IGF binding proteins, which regulate the bioavailability of insulin-like growth factors (IGFs). We also found two other MMPs known to cleave IGF binding proteins, MMP-1 and MMP-2, as well as ADAM12, to be significantly upregulated. While the failure of MMP inhibitors in clinical trials has been disappointing [106], our results indicate that MMPs continue to be attractive therapeutic targets for PC.

#### Tumor tissue heterogeneity

Most cancers are believed to originate through of a process of Darwinian evolution occurring among the cells within the microenvironments provided by the tissues of a multicellular organism. It has become increasingly clear that this process can give rise to tumor tissue heterogeneity [107], with distinct populations of cancer cells predominating in pancreatic and other tumors [16] [108]. For example, this also provides a mechanism for the development of drug resistance, whereby a minor drug resistant subclone in the original tumor becomes dominant after treatment [109]. In this context it is possible that for the datasets in our study, the number of cancer genomes sampled was higher than the number of patient samples. The task of identifying and validating diagnostic and prognostic biomarkers is likely to be complicated by the existence of tumor heterogeneity.

#### Sample heterogeneity

Most microarray datasets specifically cited the patient samples as being from either pancreatic adenocarcinoma (PAC) or pancreatic ductal adenocarcinoma (PDAC) (Table 1). These samples could still contain contaminants from the desmoplasia, particularly in studies where microdissection was not used (Table 1), contributing to sample heterogeneity. If significant contamination of tumor samples from immune components of the desmoplasia has occurred, it can have an impact on the association we found by IPA analysis between PC and inflammation/immune mechanisms. It should be noted that there is also support for an association between PC and inflammation from the literature (which we have cited previously). A second source of sample heterogeneity is the type of PC. One study (Pei Pancreas) did not specifically mention PAC or PDAC under the dataset summary, and thus could conceivably contain samples from other types of PC, although PDAC accounts for over 90% of the cases of PC.

The broad concordance observed between the microarray datasets (see Congruency between microarray datasets) suggests that issues related to sample heterogeneity (as well as other sources of variation between the microarray datasets) were not a major complicating factor in this meta-analysis. This observation also strengthens the case for investigating differentially regulated genes as putative biomarkers for PC.

## Conclusions

Meta-analysis of multiple microarray datasets can yield more reliable and comprehensive results than using a single dataset, because the former has increased statistical power and generalizability. In the present study, we performed a meta-analysis of nine PC datasets and identified 827 genes that are significantly upregulated in pancreatic cancer. The two most important biological networks associated with these genes have TGFB1 and NF-κB as major hubs. A pathway analysis indicates that PC is significantly associated with inflammation and immune mechanism.

Among the list of candidate cancer genes uncovered by this study are four highly expressed genes not previously associated with PC, and twenty-six kinase genes. Kinases have been attractive targets in combating cancer, and in fact, effective therapeutics have already been developed for several kinases in our list. Importantly, this study also revealed potential biomarkers for pancreatic cancer. Such biomarkers are in urgent need, given the poor prognosis after (the normally late) diagnosis of PC. Towards this end, we have also developed a putative genetic signature for hypomethylated genes in PC. The identification of candidate cancer genes and putative biomarkers for pancreatic cancer provide new opportunities for early diagnosis and treatment of PC.

## Supporting Information

Table S1 **The 827 genes significantly upregulated in pancreatic cancer.**
(XLSB)

Table S2 **The most significant biological functions and diseases associated with the 827 upregulated genes.**
(XLS)

Table S3 **The five most significant pathways associated with the 827 upregulated genes.**
(XLS)
